# Assessing the consistency of flammability indices across field and laboratory experimental tests for some Moroccan forest fuels

**DOI:** 10.1371/journal.pone.0345668

**Published:** 2026-04-02

**Authors:** Salaheddine Essaghi, M’hamed Hachmi, Sabrine Laajali, Mohamed Chikhaoui, Allal Hamouda

**Affiliations:** 1 Department of Natural Resources and Environment, Institut Agronomique et Vétérinaire Hassan II, Rabat, Morocco; 2 Department of Forestry Development, Ecole Nationale Forestière d’Ingénieurs, Salé, Morocco; 3 Formerly of Ecole Nationale Forestière d’Ingénieurs, Salé, Morocco; 4 Departement of Applied Statistics and Computer Science, Institut Agronomique et Vétérinaire Hassan II, Rabat, Morocco; Muhammad Nawaz Sharif University of Engineering and Technology, PAKISTAN

## Abstract

Flammability is evaluated differently depending on the plant level (whole plant, shoot, twig) and the scale of fire experiments (field versus laboratory). Inaccuracies in fire risk prediction highlight a standardisation problem, which requires comparing the flammability of the same individuals tested through contrasting experimental tests. The samples were collected from a forest site known for its high fire recurrence. Five Mediterranean tree and shrub species were tested in both laboratory and field-scale experiments, selected from the most commonly used methods, and conducted at the twig and shoot levels, respectively. In both tests, *Pinus canariensis* proved to be the most flammable species, while *Quercus suber* and *Cistus salviifolius* ranked second and fourth in flammability. The third position was taken by *Arbutus unedo* for shoot flammability and by *Pistacia lentiscus* for twig flammability. However, the shoot flammability test stood out because of its distinction between the moderately flammable group of species (*Q. suber*, *A. unedo* and *C. salviifolius*) and the least flammable group (*P. lentiscus*), that were all less flammable than *P. canariensis*, unlike the twig test. These findings could improve the understanding of researchers and forest managers regarding plant flammability, regardless of the test or device used, provided technical resources are available, at least in the study site for the five species examined. Additional tests involving a broader range of plant species are essential for a reliable comparison of the flammability assessments examined. Further methods of flammability assessment are needed to interpret empirical rankings, especially when translating laboratory results into predictive indicators of wildfire behaviour in natural ecosystems.

## Introduction

Fuel hazard assessment is crucial for preventing wildfires [1]. The flammability rating helps evaluate fire risk [2,3] and guides the selection of landscaping plants and fire-resistant species suitable for afforestation as part of a forest fire management plan [2–6]. Additionally, many studies on flammability aim to identify fire-adapted plant traits and how species contribute to fire-vegetation feedback. An accurate and realistic flammability assessment is also essential to enhance the accuracy of fire behaviour prediction models [1,6,7] and to manage wildfires efficiently [2,7–10].

The flammability of plant species remains a debated topic, especially regarding the standardisation of testing procedures and the proper measurement of flammability [11–15]. Each plant level tested aims to evaluate flammability as accurately as possible, despite the lack of standardisation [9,13]. Field-scale experimental tests, although the closest to natural conditions [11,13] and more suitable for assessing crown fires [16,17], are still underused [13,18] mainly due to requirements for authorisation, safety issues, and costs [19–22], as well as the challenges of measuring flammability in wildland fires [12,19].

Laboratory-scale flammability experimental tests are the most commonly used tests [23] as a convenience during sampling and fire tests [3,16,17,24]. These tests are based on fire experiments conducted at different levels of plant and use, therefore, using either the whole plant, shoots, twigs, or leaves, mostly using static heat flux [9,11,18,23] or dynamic (increasing) heat flux [25]. Some of these laboratory studies utilise ground samples [26–29], which are criticised for not accounting for the fuel structure and for conducting combustion tests under conditions that differ from natural conditions [9]. Such experiments alter leaf physical or biochemical traits and are seen as blurring patterns in the field [12]. However, other laboratory studies use undisturbed samples of leaves and leafy branches [6,19,20,22,23,30–32]. In this sense, laboratory-scale fire experiments using samples of terminal twigs with their leaves, such as those initiated by Valette [33] – also known as twig flammability –, which have been widely replicated by many authors [6,19,20,22,30–32,34], are now considered the most commonly used flammability assessment method [23]. Beyond conventional metrics such as time-to-ignition (TI), maximum flame height (FH) or combustion time used in twig flammability, several studies emphasise the relevance of thermophysical parameters like the heat release rate (HRR) and the effective heat of combustion (EHC), which provide a more physically meaningful estimate of combustibility and the intensity and propagation of fire [26–28,35]. However, the heat release rate (HRR) can be adequately replaced by specific pyric properties, such as the time-to-ignition (TI) and duration of flaming [35]. In addition, the heat release rate (HRR), being the basis for calculating the effective heat of combustion (EHC) [36], the duration of burning and time-to-ignition can also be proxies for the latter.

However, Fernandes and Cruz [37] argue that the suitability of some of these laboratory-scale flammability tests is questionable when scaling up to predict canopy flammability in the field. The fuel bed packing ratio (or compactness), another important flammability trait that affects fire behaviour [38–42], is not considered in laboratory-scale experiments using fuel elements [37]. Nevertheless, fire effects occur across various scales; fire behaviour can be assessed using different flammability metrics, which require testing on both cut and undisturbed samples [43,44]. Therefore, not only are fuel bed and field burning tests important, but individual species flammability tests also play a key role in predicting fire behaviour, as the ignitability of individual leaves has been correlated with several flammability components tested in fuel beds, including the rate of fire spread [16]. In fact, many fire behaviour prediction systems rely on the individual contribution of each species’ flammability to fire behaviour [45,46], which is modelled to address the inadequate weighting of fuel characteristics and their interactions [37]. Additionally, fire experiments on small samples in the laboratory assist in assessing realistic plant flammability, since natural selection and genetic control work on traits at the individual level, requiring standardised flammability measurements on leaves or small branches under controlled laboratory conditions [44]. Flammability metrics for cut material should correspond with those for undisturbed material in the field to enable accurate scaling up and reflect real-world conditions [37]. Therefore, by measuring flammability on cut samples (small samples), each flammability trait is weighted according to its contribution to fire risk, making this type of flammability testing valuable for calibrating fire behaviour prediction models [44]. Laboratory-scale flammability experimental tests allows us to improve both our understanding of plant evolutionary processes and modelling tools for simulating fire hazard and behaviour [47].

In light of the rising global occurrence of crown fires, it is crucial to consider canopy fuel characteristics [48,49]. In this context, a method based on field-scale fire experiments on whole shoots, known as shoot flammability [50,51], serves as an alternative to burning entire plants [11,15,51] and acts as a substitute for field burnings [7,11,15]. These fire experiments measure the maximum temperature reached during combustion (MT), the duration of burning (BT), and the length of the burnt portion of the shoot (BL), all conducted in the field using a standardised grill suggested by Jaureguiberry et al. [50]. Shoot flammability has been employed by various authors (e.g., [7,15,38–42,52]). Besides its benefit of maintaining the natural architecture of plant shoots [7], shoot flammability is considered the most crucial consideration for canopy flammability because it pertains to the structure of individual crowns, which is inadequately addressed in other flammability-testing methods [7,11,15]. It has also been shown that shoot flammability provides a flammability ranking highly correlated with that determined by expert opinion based on field observations [7]. Shoot flammability, which counts for 15% of FLAMIT’s plant flammability database, allowed for more realistic values of plant flammability, because it takes into account (at least partially) the effect that plant architecture has on fuel connectivity as well as the airflow through the fuel, both of which significantly influence flammability [7,47,53]. Additionally, the parameters used for both shoot and twig flammability are applicable across field and laboratory scales [35,23,54–56].

However, fire experiments conducted at both field and laboratory scales can produce significantly different estimates of flammability [24], leading to conflicting predictions from fire behaviour systems [9]. These discrepancies may arise from differences in measurement methods, tools, sampling techniques [11,38], or the specific variables considered by each approach [14]. Globally, this issue is often linked to the scale of the experiment [57,58] or the characteristics of the fuel [59]. For example, Molina et al. [59] observed either similarities or differences in cork burning parameters, depending on corkback roughness, using both laboratory and field fire tests. Recognising this debate and contributing to the collective effort to better understand plant flammability and improve fire behaviour forecasts, it is essential to seek repeatability and metrics that can be scaled from laboratory to the field, ultimately standardising flammability testing methods [37]. To achieve this, we compare plant flammability using the most contrasting experimental tests—specifically, contrasting scales (lab versus field) and plant parts (whole shoot or at least a 70 cm shoot versus a 1 g terminal twig with leaves or needles): (i) shoot flammability and (ii) twig flammability. This comparison aims to establish correlations between field and laboratory parameters, thereby increasing the repeatability of fire experiments. These tests will subsequently be performed based on available technical resources, with the goal of identifying shoot-based flammability that corresponds to twig flammability, facilitating scaling up and reflecting real-world conditions. Alam et al. [15] compared existing data from various authors who employed different flammability tests, including shoot flammability [7] and leaf-level flammability measured via a muffle furnace [24]. However, factors such as site effects that influence pyric properties [5,19,60,61], in addition to the repeatability of experimental procedures, must also be considered. To address these issues, we neutralise these factors by testing samples from the same individual plants. We anticipate that this comparison will yield similar rankings regardless of the specific flammability test used.

## Materials and methods

### Study site

The chosen site was situated in the Izarene forest, near Ouezzane city, in the Western Rif Mountains of Morocco, Northwest Africa (N34°48’22.7”; W5°30’13.2”; 421 m). This geographical area overlooks the Mediterranean Sea to the north and the Atlantic Ocean to the west. The site’s slope is 30%. It was selected because it hosts the most prevalent Mediterranean forest fuels and is part of a region known for frequent and severe wildfires. The average fire size in the region is 1027 ha, and 45.3% of Morocco’s burned area occurs in the western Rif Mountain region [62–64]. At the nearest meteorological station (Ouezzane), long-term climate data are as follows: mean annual rainfall, 751 mm; minimum temperature, 5.5°C; and maximum temperature, 34.3°C (www.climate-data.org; Site visited on 12 December 2022).

### Species selection and sampling

The samples examined were collected on 17 March 2020 from five tree and shrub species. No collection permit was required. The Izarene forest belonged to the Moroccan Department of Waters and Forests, Ministry of Agriculture, Fisheries, Rural Development, Waters and Forests in Morocco. On the collection day, the temperature ranged from 12.5 to 19.2°C, while the relative humidity varied between 59.5% and 63.2%, and the wind speed was between 1.3 and 1.5 m/s. The plant individuals sampled were healthy-looking, adult, and randomly selected from each species at locations with different solar irradiances to reduce the potential effect of solar irradiance intensity. The tree species studied included *Quercus suber* (cork oak) and *Pinus canariensis* (Canary Island pine). The shrub species were *Arbutus unedo* (strawberry tree), *Cistus salviifolius* (sage-leaved rockrose) and *Pistacia lentiscus* (mastic tree). All species were chosen based on their abundance in the forests of the Western Rif. The samples were collected from the same site and same plant individuals in controlled conditions that avoids harmful variables, including interspecific and intraspecific effects.

### Field-scale flammability test using shoot samples on the grill

The flammability experimental test used to assess flammability through field-scale fire experiments involving large fuel samples is as described by Jaureguiberry et al. [50]. This experiment is necessary to estimate the “shoot flammability”. For each species, six samples consisting of 70 cm-long terminal branches were collected from six plant individuals of different ages, immediately before the fire tests.

For each species and sample, three parameters were considered: the maximum temperature reached during combustion (MT, in degrees Celsius), the duration of burning (BT, in seconds), and the length of the burnt portion of the shoot (BL, in centimetres). The burning rate (BR) was calculated by dividing the length of the burnt portion of the shoot (BL) by the duration of burning (BT). Conversely, the burnt biomass percentage (BB, expressed as a percentage) was estimated visually and practically by measuring the length of the shoot portion that did not burn after spontaneous fire die-off. As described in Jaureguiberry et al. [50], a value was assigned to the burnt biomass percentage (BB) based on the class of percentage of burnt biomass. This value corresponded to one of six categories: 1 ≤ 1%, 2 = 1–10%, 3 = 11–25%, 4 = 26–50%, 5 = 51–75%, and 6 = 76–100%.

For each species and sample, the maximum temperature during combustion (MT), the burning rate (BR), and the percentage of burnt biomass (BB) were converted into standardised scores (respectively called MTscale, BRscale, and BBscale), ranging from 0 to 1, by dividing each by the corresponding reference values proposed by Jaureguiberry et al. [50]. The reference values are the maximum temperature during combustion (MT), set at 500°C; the burning rate (BR), which is 1 cm/s; and the burnt biomass percentage (BB), set at 6.

For each sample, the shoot flammability index (FLS) was calculated as the sum of three standardised components (Equation 1):


$${FL}_{S}={MT}_{scale}+{BR}_{scale}+{BB}_{scale}$$ (1)

where MT_scale_: standardised score of the maximum temperature reached during the combustion; BRscale: standardised score of the burning rate, and BB_scale_: standardised score of the burnt biomass percentage.

**Fig 1:**
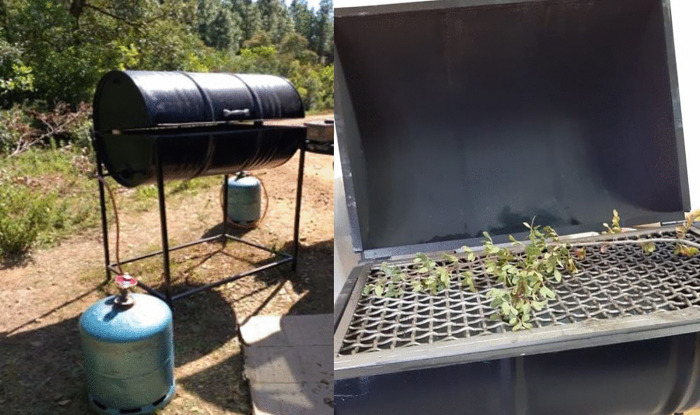
Photographs of the standardised grill used for field shoot flammability testing.

This composite index ranges from 0 to 3. Fire experiments were carried out at the site using the same device described by Jaureguiberry et al. [50] (Fig 1). It comprises a transportable, customised device with a core consisting of a metal barrel cut in half, positioned horizontally and mounted on four metal legs. The device also features three burners, a grill, and a blowtorch. Each burner and the blowtorch are connected to a butane gas cylinder. Temperature was measured using a Testo 176 T4 data logger (measuring range: −200–1000°C; measurement cycle: 1 second to 24 hours (freely selectable)) linked with a Testo Type-K thermocouple probe. The thermocouple was attached along the grill over a length of 45 cm [40].

The device was proposed by Jaureguiberry et al. [50] and later described by Pérez-Harguindeguy et al. [51] for assessing shoot flammability in the field. It consists of a standardised grill equipped with a blowtorch, thermocouple probe and data logger. Photos: Sabrine Laajali and Mohammed Rhaz. The two butane gas bottles shown in the photographs supply, respectively, the grill burners and the blowtorch.

### Laboratory-scale flammability test using twig samples on the epiradiator

Flammability was evaluated through a laboratory-scale fire test using small samples. The experimental test employed is the one proposed by Valette [33], revised by Hachmi et al. [34] and further by Essaghi et al. [6]. It involves calculating a flammability index based on fire tests conducted on 1 g samples of terminal branches with their leaves or needles. This test is essential for estimating the “twig flammability”.

For each species, 42 g of plant material was collected from three to four mature individuals [6]. The samples were taken from the same individuals involved in the shoot-flammability experimental test. They were placed in sealed plastic bags and transported in a thermally insulated box with ice, as described in Behm et al. [60].

**Fig 2:**
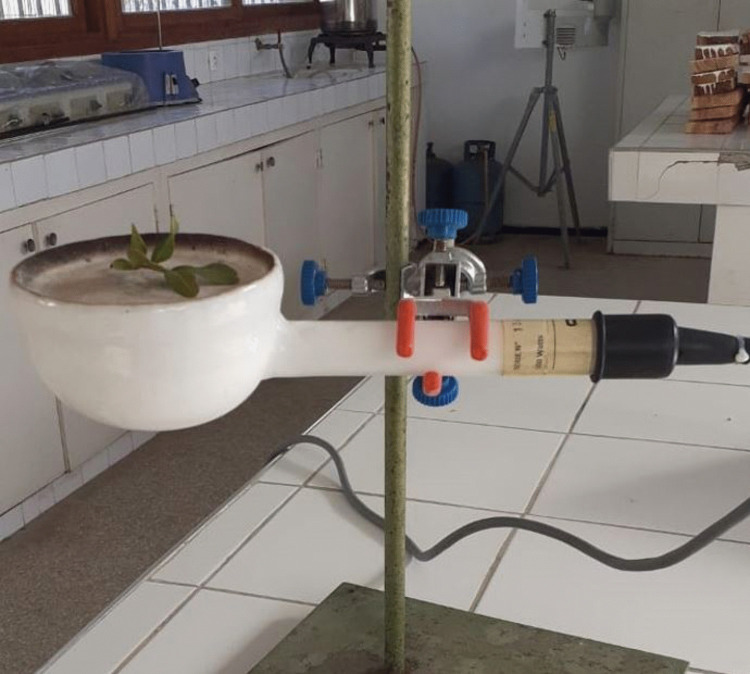
Photograph of the epiradiator used for twig flammability testing of fuel species in the laboratory.

For each species, plant material was divided into two groups. The first group, intended for fire tests, consisted of 36 samples of 1 g each, while the second group was reserved for moisture content (MC) tests [6]. Each sample in the first group was placed on the well-heated ceramic surface of the epiradiator (Fig 2). Time-to-ignition (TI) was recorded in seconds using a stopwatch and maximum flame height (FH) was measured in centimetres with a vertical graduated ruler [33].

The epiradiator is mounted on a metal support that holds a ruler used for measuring the maximum flame height, as described by Valette [33]. The samples are placed on the ceramic plate of the epiradiator, where they are heated before ignition and a stopwatch records the time-to-ignition. Photo: Mohammed Rhaz.

For each species, the twig flammability index (FLT) was calculated using Equation 2 [6], based on the previously mentioned 36 replicates.


$${FL}_{T}=\text{3}\times \frac{\text{60}-\text{TI}}{\text{12}.\text{5}+\text{TI}}\times e^{{\left(\frac{FH}{FH+\text{4}\text{0}}\right)}^{\text{2}}}$$ (2)

where TI is time-to-ignition and FH: maximum flame height. FH should change from 0 to 40 cm and TI from 0 to 60 s. The negative test is declared (FL_T_ = 0) when TI = 60 s and FH = 0 cm. 12.5 was chosen since it represented the inferior limit of TI variation range according to [33]. To introduce a rating out of 20 points, the equation should be multiplied by 3. The exponential function was chosen so that when the flame does not exist, FL_T_ is not affected (*e*^0^ = 1).

A ranking of species has been established based on the twig flammability index (FLT).

Fire tests were conducted using a Quartzalliance epiradiator 500 W 534 Rc 2, as described by Valette [33] (Fig 2). The radiant disc emits a steady heat flux of around 600°C. A pilot flame is positioned 4 cm above the centre of the disc, while a millimetre ruler is placed perpendicular to the ceramic surface to measure the maximum flame height (FH). The epiradiator and pilot flame are kept under a hood to prevent draughts that could interfere with ignition and burning process [65].

In the second group, three 2-g samples were selected simultaneously with the samples chosen for fire experiments and placed in the oven at 60°C for 72 hours. The samples were weighed to obtain the ovendry weight [60,66] in order to determine the moisture content (MC) values during fire tests calculated as a percentage of oven-dry weight using Equation 3 [63].


$$\text{MC}=\left(\frac{Fresh\;weight-Dry\;weight}{Dry\;weight}\right)\times \text{1}\text{0}\text{0}$$ (3)

All weights were measured using a Kern PCB 1000−1 balance with an accuracy of 0.1 g.

### Statistical analyses

Species data were analysed to assess the effects of the species, using Tukey's tests for pairwise comparisons to check the significant differences between species for each studied parameter (H_0_, no significant difference between the species). For each test, H_0_ is rejected when *p*-value<0.05. The alternative hypothesis was “H_1_, there is a significant difference between species”.

To highlight the flammability parameters for both fire experiment scales, relationships between moisture content (MC), the shoot flammability index (FL_S_) and the twig flammability index (FL_T_), along with their metrics, were examined using Pearson’s correlation matrix to determine the correlation coefficient *R*. Regressions between the shoot flammability index (FL_S_) and the twig flammability index (FL_T_) were also explored.

Discriminant analysis was employed to identify the metrics that best predict the flammability ranking obtained from each flammability-testing experiment and to facilitate the comparison of both classifications. Principal component analysis (PCA) was conducted on the shoot, followed by the twig flammability traits of the five species, to explore the relationships between these various flammability traits. To control for any differences in flammability, the shoot and twig flammability rankings were compared using Spearman’s rank-order correlation, which assessed the strength of the association between two ranked variables (H_0_, no association between the two variables).

All analyses were conducted using the IBM Statistical Package for the Social Sciences (SPSS version 23) (SPSS Inc., Chicago, IL, USA).

## Results

### Field-scale flammability test using shoot samples on the grill

The mean values of shoot flammability metrics measured during the field-scale experiments and the calculated parameters (BB, BR, and FLS) are presented in Table 1. These experiments showed that *P. canariensis* shoots had the highest mean values across all parameters studied. Specifically, the duration of burning (BT) had a mean of 1091.7 s, the maximum temperature reached during combustion (MT) averaged 211.2°C; and the length of the burnt portion of the shoot (BL) averaged 62.8 cm, placing *P. canariensis* at the top of the flammability scale (FLS = 1.52). However, there was no significant difference between the BT values of *P. canariensis* and *Q. suber* (p = 0.130 at α = 0.05). *Cistus salviifolius* shoots had the highest mean BR-value (0.22 cm·s ⁻ ¹), but it was not significantly different from the BR-values of *A. unedo* and *Q. suber* (p = 1.00). The fire tests also indicated that *P. lentiscus* shoots were significantly the least flammable (p = 0.001 with *A. unedo*, *P. canariensis* and *Q. suber* and p = 0.04 with *C. salviifolius*); FLS = 1.03). For this species, all measured parameters showed the lowest values, although these were not significantly different from those of most other species (p = 0.055 with *P. canariensis*, p = 1.00 with the rest of species for BT; p = 0.524 with *Q. suber*, p = 0.90 with *A. unedo*, p = 0.96 with *C. salviifolius*, but p = 0.001 with *P. canariensis* for MT; p = 0.081 with *A. unedo* and p = 0.769 with *C. salviifolius*, but p = 0.001 with *P. canariensis* and p = 0.028 with *Q. suber* for BL) (Table 1). The comparison of FLS-values among the examined species, using one-way ANOVA and Tukey’s multiple comparison tests (95% confidence level), revealed a highly significant statistical difference, forming three significance levels (Table 1). *Pinus canariensis* was the only species in the “Most flammable” category, while *P. lentiscus* was the sole species in the “Least flammable” category. *Arbutus unedo*, *C. salviifolius*, and *Q. suber* were classified as “Moderately flammable” fuels.

**Table 1:** Shoot flammability parameters*.

Species	BT (s)¤	MT (°C)¤	BL (cm)¤	BB (%)¤	BR (cm.s^-1^)¤	FL_S_¤	Flammability class
***Pinus canariensis***	1091.7 ± 541.9a	211.2 ± 14.8a	62.8 ± 3.6a	89.6 ± 5.1a	0.09 ± 0.03a	1.52 ± 0.01a	Most flammable
***Quercus suber***	356.5 ± 153.3ab	174.2 ± 3.4b	42.8 ± 7.0b	61.1 ± 10.0b	0.16 ± 0.03ab	1.31 ± 0.04b	Moderately flammable
***Arbutus unedo***	260.1 ± 24.9b	168.8 ± 1.1b	38.8 ± 5.0bc	55.5 ± 7.1bc	0.16 ± 0.03ab	1.27 ± 0.06b	
***Cistus salviifolius***	155.4 ± 31.5b	167.6 ± 1.4b	29.8 ± 1.6bc	42.6 ± 2.3bc	0.22 ± 0.04b	1.22 ± 0.04b	
***Pistacia lentiscus***	259.2 ± 27.7b	162.75 ± 1.7b	22.5 ± 3.2c	32.1 ± 4.6c	0.09 ± 0.01a	1.03 ± 0.04c	Least flammable

*The p-values obtained between every two species for each parameter studied are displayed in Table 1 of the Supporting Information section S1 Table; ¤Within a column, the means followed by the same letter are not significantly different at α = 0.05 in the Tukey’s test. Duration of burning, the maximum temperature reached by the burning shoot, length of the burnt portion of the shoot, burnt biomass percentage and burning rate measurements and shoot flammability index are displayed per species and with ± standard error. Within a column, the species followed by the same letters are not significantly different (p > 0.05) in Tukey’s pairwise comparison.

BT: burning time; MT: the maximum temperature reached; BL: burnt length; BB: burnt biomass; BR: burning rate; FL_S_: shoot flammability index.

**Fig 3:**
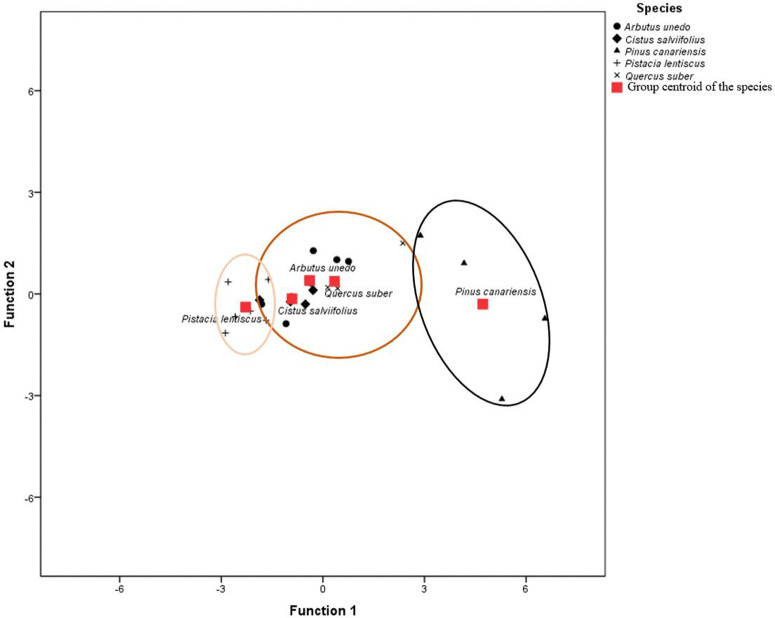
Classification of the five Mediterranean species, ordered from the most flammable (darkest outline) to the least flammable (lightest outline), based on the shoot-flammability experimental test.

The coefficients in the discriminant functions were chosen based on stepwise analysis that maximised inter-group variance. The linear form of the functions was maintained due to the normality of residuals; however, non-linear modelling could be explored in future studies to capture potential interactions. According to the linear discriminant analysis, the best predictor of the species flammability ranking was MT (standardised canonical discriminant function coefficient of 0.595), while BT had the least explanatory power of the species flammability ranking with a coefficient of 0.295. With a high eigenvalue of 6.004, a statistically significant Wilk’s lambda, and a value of 0.118 (close to 0; p < 0.0001), the canonical discriminant function effectively explained the species flammability ranking, indicating a good fit of the prediction model. The expressions for functions 1 and 2 shown in Fig 3 are: Function 1: D = −0.002BT + 0.13MT + 0.068BL-24.278; Function 2: D = −0.053MT + 0.068BL + 6.747, where D is the discriminant score. The flammability classes identified in the distribution of scores (Fig 3) correspond with the three classes noted following Tukey’s pairwise comparison test, sharing the same class composition.

The observations show the predicted scores of the canonical discriminant function for each species after linear discriminant analysis. Function 1: D = −0.002BT + 0.13MT + 0.068BL-24.278; Function 2: D = − 0.053MT + 0.068BL + 6.747. D: discriminant score; BT indicates the duration of burning; MT signifies the maximum temperature during combustion and BL refers to the length of the burnt part of the shoot. The group centroids of species within the same class are closer than those of different classes, confirming the results of Tukey’s pairwise comparison test. The classification identified three flammability classes: the most flammable (*Pinus canariensis*), a moderately flammable group including *Arbutus unedo*, *Cistus salviifolius* and *Quercus suber* and the least flammable group comprising *Pistacia lentiscus*.

### Laboratory-scale flammability test with twig samples on the epiradiator

The mean values of twig flammability parameters measured through laboratory-scale fire experiments are shown in Table 2. *Pinus canariensis* twigs were significantly the most ignitable among all species examined (*p* = 0.001), with a time-to-ignition (TI) of 5.17 s. This species also produced, on average, the tallest flames, with a maximum flame height (FH) of 17.69 cm, although this was not significantly different from the values recorded for *Q. suber* (*p* = 0.073). Therefore, *P. canariensis* twigs were significantly the most flammable among all species studied, with an FLT-value averaging 10.38 (*p* = 0.001) (Table 2). *Arbutus unedo* twigs exhibited the lowest mean FLT-value (6.77), but they did not significantly differ from the other species (*p* = 0.067 > 0.05 with *Q. suber*, *p* = 0.118 > 0.05 with *P. lentiscus* and *p* = 0.282 > 0.05 with *C. salviifolius*), except *P. canariensis*, for which the difference was significant (*p* = 0.001 ≤ 0.05) (Table 2). The comparison of FLT values across the species, using one-way ANOVA and Tukey’s pairwise comparison tests (95% confidence level), revealed a highly significant statistical difference, dividing the species into two groups. *Pinus canariensis* was the only species classified as “Most flammable,” while all the other species fell into the “Least flammable” category.

**Table 2:** Twig flammability parameters*.

Species	TI (s)¤	FH (cm)¤	FL_T_¤	Flammability class
***Pinus canariensis***	5.17 ± 0.32a	17.69 ± 0.54a	10.38 ± 0.25a	Most flammable
***Quercus suber***	9.43 ± 0.40b	15.01 ± 0.85ab	7.59 ± 0.20b	Least flammable
***Pistacia lentiscus***	9.47 ± 0.45b	13.63 ± 0.62b	7.51 ± 0.21b	
***Cistus salviifolius***	8.83 ± 0.44b	2.63 ± 0.43c	7.39 ± 0.21b	
***Arbutus unedo***	10.40 ± 0.53b	2.68 ± 1.02c	6.77 ± 0.23b	

*The p-values obtained between every two species for each parameter studied are displayed in Table 2 of the Supporting Information section S1 Table; ¤Within a column, the means followed by the same letter are not significantly different at α = 0.05 in the Tukey’s test. Time-to-ignition, maximum flame height measurements and calculated twig flammability index are displayed per species and with ± standard error. Within a column, the species followed by the same letters are not significantly different (p > 0.05) in Tukey’s pairwise comparison.

TI: time-to-ignition; FH: maximum flame height; FL_T:_ twig flammability index.

**Fig 4:**
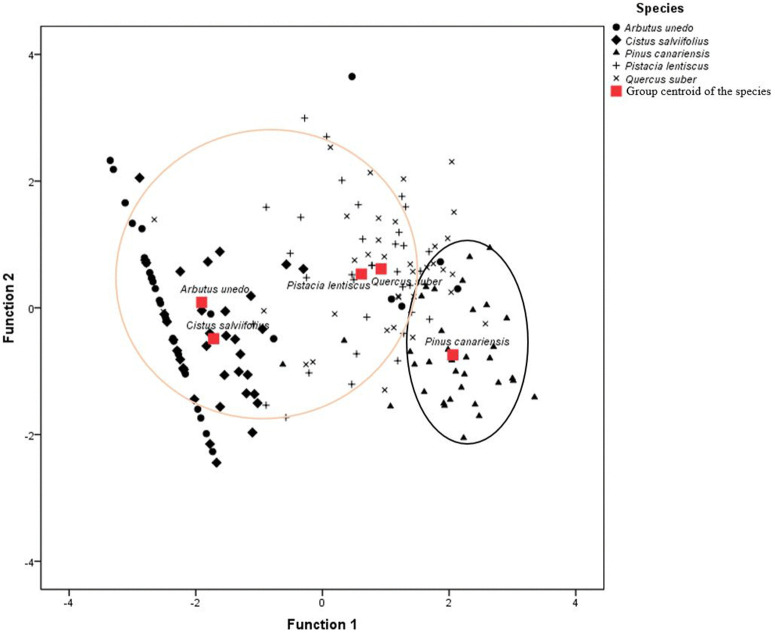
Rankings of the five Mediterranean species from the most flammable (darkest outline) to the least flammable (lightest outline) species based on the twig-flammability experimental test.

This classification encounters that obtained by the linear discriminant analysis (Fig 4), where three classes can be distinguished from the most to the least flammable. The first class was made up of *P. canariensis* only, the second consisting of *P. lentiscus* and *Q. suber* and the third one consisting of *A. unedo* and *C. salviifolius* (Fig 4). This classification fits well with the FL_T_ values, which seemed to be the highest for *P. canariensis*, while for the rest of the species, they were close to each other’s, though *A. unedo* and *C. salviifolius* seemed to be less flammable than *P. lentiscus* and *Q. suber*.

The observations show the predicted scores of the canonical discriminant function for each species following linear discriminant analysis. Function 1: D = −0.128TI + 0.22FH-1.164; Function 2: D = 0.364TI + 0.071FH-3.889; D: discriminant score; TI: time-to-ignition; FH: maximum flame height. The classification identified two flammability groups: the most flammable group (*Pinus canariensis*) and the less flammable group, which includes all other examined species: *Arbutus unedo*, *Cistus salviifolius*, *Pistacia lentiscus* and *Quercus suber*. This classification is based on ANOVA followed by Tukey’s pairwise comparison test and linear discriminant analysis.

The standardised canonical discriminant function coefficient for FH was 0.951, while for TI, this coefficient was 0.332. Therefore, FH was the best predictor of the species’ flammability ranking in the twig-flammability experimental test. With an eigenvalue of 2.489 (greater than one) and a statistically significant Wilk’s lambda value of 0.220 (close to zero and p < 0.0001), the canonical discriminant function effectively explained the species’ flammability ranking (indicating a good fit of the prediction model). The expressions for functions 1 and 2, shown in Fig 4, are as follows: Function 1: D = − 0.128TI + 0.22FH-1.164; Function 2: D = 0.364TI + 0.071FH-3.889, with D representing the discriminant score.

The dampest samples were from *A. unedo* (236.11%) and *C. salviifolius* (213.46%), whose MC-values were not significantly different (p = 0.770), but were significantly higher than those of *Q. suber* (139.17%), *P. canariensis* (87.40%) and *P. lentiscus* (87.36%) (p < 0.001). The MC-values of the latter species were not significantly different from each other (p ≥ 0.130).

### Comparison of flammability between twig and shoot parameters

Both shoot- and twig-flammability experimental tests resulted in a common outcome: *P. canariensis* was classified alone in the ‘Most flammable species’ category, while the other examined species were found to be less flammable. However, for these latter species, shoot and twig-flammability tests produced two different classifications. The results from the shoot-flammability tests provided more detail by differentiating between species less flammable than *P. canariensis*, whereas the twig-flammability test grouped all species except *P. canariensis* into a single category.

**Fig 5:**
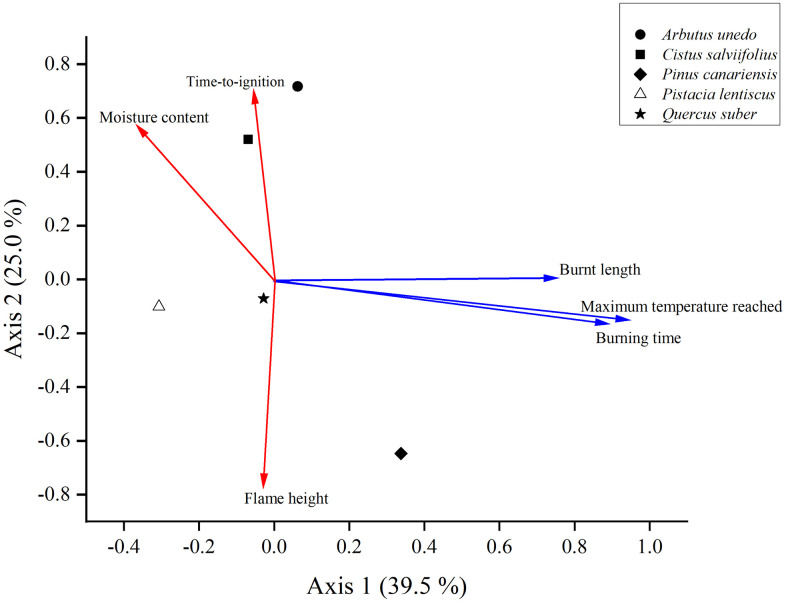
Principal component analysis (PCA) of shoot flammability traits (blue vectors: maximum temperature reached (MT), burning time (BT) and burnt length (BL)) and twig flammability traits (red vectors: time-to-ignition (TI), flame height (FH) and moisture content (MC)).

Principal component analysis (PCA) of the plant flammability data indicated that twig flammability parameters, in addition to MC, were orthogonal to shoot flammability parameters (Fig 5). Consequently, TI and FH (twig flammability parameters) showed no correlation with MT, BT, and BL (shoot flammability parameters). However, TI and FH were correlated with MC. These findings are also supported by the data in Table 3. PCA was applied as an exploratory analysis to visualise relationships among flammability traits at the species level. The first two axes explained 39.5% and 25% of the total variance, respectively, with axis 1 mainly associated with BT, MT and BL and axis 2 with TI and FH. Axis 2 opposed TI and FH. MC is in the slightest degree associated with both axes. Given the small number of species, PCA is interpreted descriptively.

**Table 3:** Pearson’s correlation matrix regarding the parameters burning time (BT), maximum temperature reached (MT), burnt length (BL), time-to-ignition (TI), maximum flame height (FH) and moisture content (MC).

	BT	MT	BL	TI	FH	MC	FL_S_	FL_T_
**BT**		0.883*	0.454*	−0.365	0.351	−0.367	0.441*	0.504**
**MT**	0.883*		0.585*	−0.335	0.423	−0.391	0.664**	0.471*
**BL**	0.454*	0.585*		−0.190	0.356	−0.212	0.907**	0.280
**TI**	−0.365	−0.335	−0.190		−0.473*	0.517*	−0.144	−0.959**
**FH**	0.351	0.423	0.356	−0.473*		−0.925**	0.013	0.451**
**MC**	−0.367	−0.391	−0.212	0.517*	−0.925**		−0.078	−0.609*
**FL**_**S**_	0.441*	0.664**	0.907**	−0.144	0.013	0.078		0.180
**FL**_**T**_	0.504**	0.471*	0.280	−0.959**	0.451**	−0.609*	0.180	

*Significant correlation at α = 0.05.

**Significant correlation at α = 0.01.

All shoot flammability parameters were positively loaded on PCA axis 1 (MT: 0.941; BT: 0.885 and BL: 0.752), while twig flammability parameters had either negative (FH: −0.796) or positive (TI: 0.702) loadings on the second axis. MC was positively loaded on PCA axis 2 (0.571). At the shoot level, species with a high PCA score across all three traits on the first axis and a low score on the second axis were more flammable. This means that species recording high MT, BL and BT values (positive loadings on axis 1) and low TI (positive loading on axis 2) but high FH – as FH had negative loading on axis 2 (low PCA score on axis 2), as shown in Fig 5 – were more flammable (Tables 1 and 2). In other words, species considered highly flammable according to shoot flammability showed low TI (more ignitable) and high FH (high flame) indicating more fire spread, *i.e.,* this species is also highly flammable according to twig flammability. This is clearly the case of *P. canariensis*. Conversely, at the twig level, species with a low PCA score for flame height on the second axis were more flammable. This means that species with high FH, as FH had negative loading on axis 2 (low PCA score on axis 2), were more flammable. *Pinus canariensis* fits well with this analysis and vice versa *Arbutus unedo* and *C. salviifolius*, which had a high PCA score for FH on axis 2 (Fig 5) were less flammable (Table 2). These results align with the classification derived from ANOVA, Tukey’s pairwise comparison test and linear discriminant analysis (Fig 3 and 4 and Tables 1 and 2).

Each point indicates a species’ average score. The PCA of the plant flammability data revealed that twig flammability parameters and MC were orthogonal to shoot flammability parameters (Fig 2). Consequently, TI and FH were not correlated with MT, BT, or BL. However, TI and FH were correlated with MC.

According to Spearman’s rank-order correlation, the rankings obtained for shoot and twig flammability were not correlated (*ρ* = 0.235; *P* = 0.249: weak), confirming the rankings of the species studied except for *P. canariensis*. This result highlighted a significant difference in the species composition of the ‘Moderate’ and ‘Low’ flammability classes.

Overall, although each flammability experimental test (shoots or twigs) used different sets of metrics with varying scales, the flammability class was the same for three out of five species. In fact, in both tests, *P. canariensis* was the most flammable species, followed by *Q. suber*, while *C. salviifolius* ranked fourth (Tables 1 and 2). Expanding the range of species tested would help to draw more definitive conclusions about the differences in flammability observed between field and laboratory settings.

## Discussion

Most flammability components measured varied within the ranges recorded by Dent et al. [39] (MT and BB) and Wyse et al. [38] (TI and BB). In our experiments, where samples had a negligible proportion of dead material, MT values matched those with the lowest proportions of dead material tested by Dent et al. [39]. However, BB values were close to most of the values found by the latter authors, regardless of the proportion of dead material [39]. MT values in our experiment were lower than those found by Wyse et al. [38], since our samples were fresh — tested *in situ* immediately after harvest [39], unlike those used by the latter, which were dried [7,38].

The shoot-flammability experimental test has classified species into three groups, while the twig-flammability test groups all less flammable species than *P. canariensis* into a single group. In the shoot-flammability test, samples were evaluated under field conditions with MC, and the spatial arrangement of branches, twigs, and leaves—specifically bulk density—remained unchanged, as the samples were whole shoots or 70 cm-long shoots [7,11,15]. Conversely, twig flammability was assessed on 1 g samples cut from plant individuals, where the spatial arrangement of the shoots, from which the twigs were collected, inevitably changes, in addition to minor MC variations that might have occurred during transport to the laboratory. This represents a source of uncertainty when comparing field-shoot and laboratory-twig tests. However, it is important to recognise that using only single shoots or terminal twigs may not fully represent the physical compactness of a species’ fuel structure, which is a vital factor influencing fire spread potential. Fuel bed compactness affects oxygen diffusion and flame continuity; neglecting it can lead to underestimating combustibility in field conditions. As demonstrated by Fernandes and Cruz [37] and supported by various studies (*e.g*., Pausas [67], Krim et al. [68], Rothermel [69], He et al. [70] and El Houssami et al. [71]), compactness (packing ratio) and continuity are essential structural features of wildland fuels that significantly influence fire behaviour. For example, combustion time for a single needle lasts only a few seconds, whereas for a compact bed of needles, it may extend up to one minute. This emphasises the importance of fuel arrangement in fire persistence. Although the individual contribution of each species’ flammability to fire behaviour is a key input in many fire behaviour prediction systems [45,46], which aim to address limitations caused by inadequate weighting of fuel characteristics and interactions [37], this contribution was also correlated with various flammability components tested in fuel beds [16].

The decrease in MC for the samples used to estimate twig flammability—following transport from field to laboratory—likely contributes to the difficulty in detecting differences in flammability (all species except *P. canariensis* grouped in one category). Furthermore, the fine fuel content may vary between the large samples used for the shoot-flammability experimental test (70 cm-long branches) and the smaller samples (1 g-terminal twigs with leaves) used for the twig-flammability test, which could misrepresent the plant due to the higher proportion of fine fuel in twig samples compared to shoot samples [7,11,15]. Consequently, these factors influence the increased disparity observed between the two flammability tests (shoots and twigs). Since leaf physical or biochemical traits were not changed during sample preparation, the realism of the shoot-flammability test is emphasised compared to the other tests [12]. Indeed, the arrangement of leaves and twigs at the shoot level accurately mimics how fire propagates through a plant canopy, with fire spreading from twig to twig on a shoot similar to that from branch to branch in a canopy [15]. The shoot-flammability test also provides an alternative to field burnings, which face challenges such as cost, authorisations, and safety, by reducing the scale needed to assess canopy flammability while maintaining the structure of the crown [7,11,15]. Differences in heating mode and unquantified heat flux, especially for the grill used to measure shoot flammability, may partly explain discrepancies between shoot- and twig-based flammability indices.

For discontinuous fuel beds, convective heat transfer is necessary for fire spread and is related to flame dynamics [72–74]. The convective warming effects are the result of the flow of hot gases inside the flame base. This flow is subject to upward gas velocity and wind velocity. This flow creates a contact flame that preheats and ignites the unburnt fuel. The higher the flow of hot gases is, the quicker the fire spread will be, *i.e.,* convection decreases BT [75]. In our experiments, wind velocity is neutralised in the case of twig flammability by the presence of a hood protecting the epiradiator and in the case of shoot flammability by the wind protection on the grill. Convection is then carried out only by upward gas velocity. Under some conditions, convection was found to be the primary heat transfer mechanism [76]. However, heat transfer is rather performed through convective *vs*. radiative flux [77–80], as long as most heat transfer physical models (*e.g.,* FireStar3D [81], FIRETEC [82], FireFoam [71] and Wildland-Urban Interface Fire Dynamics Simulator WFDS [83]). Radiation is the main mechanism for ignition, which is considered in twig flammability using the epiradiator [47]. Hot embers and flames emit electromagnetic waves that heat the surface of nearby unburned fuel until it reaches its pyrolysis temperature, *i.e.,* the radiative flux decreases TI.

The most flammable species based on both experimental tests (shoots and twigs) was, as expected, *P. canariensis*. Many authors (*e.g*., [6,68]) have regarded this species as ‘highly flammable’. This is explained by its flammability index—which includes TI, FH, and MC [6]—and the surface area-to-volume ratio (SVR) of its needles, which is the highest among all species examined [61], thereby increasing the plants flammability [9,15,19,84]. As SVR is high, the packing ratio is also high as well as the extinction depth [75,85]. Particles with high SVR have higher surface exposed to O_2_ and heat and less internal mass to heat up to the ignition point, whilst they lose heat and MC quickly (low heat content) [85,86]. The higher SVR is, the lower the flame residence time will be [87]. In recent work by Krim et al. [68], who compared Mediterranean species using laboratory thermal metrics, *P. canariensis* was found to be approximately 22 times less effective as a fire retardant than *Phoenix dactylifera* at high irradiance, while it spread fire 15 times more than *P. dactylifera* at high irradiance and about 2.5 times more at low irradiance. Furthermore, *P. canariensis* had the lowest MC value in our experiment, which increases its flammability even under field conditions. Indeed, MC is negatively correlated with the likelihood of ignition in forest fuel beds [15,88]. This aligns with shoot flammability results found by [52]. The species’ serotiny and heat-induced seedling growth [89], which are positively associated with flammability at field scales [55], are additional factors explaining its high flammability. These findings, highlighting the comparatively high flammability of *P. canariensis*, reinforce confidence in our ranking methodology and support the external validity of our approach.

The rankings of the other species examined varied quite a bit depending on the flammability-testing experiment used. However, factors that make these species less flammable than *P. canariensis* indicate that the rankings derived from the studied experiments align with the literature, thereby increasing the credibility of our approach to evaluate plant flammability. These factors are outlined below:

(i) Relatively high MC of leaves: *P. lentiscus* [84,90]; *A. unedo*, as observed in our results and by other authors [90–92]; *C. salviifolius*, as indicated by our findings and Henaoui [93].

Conversely, based on both flammability testing experiments (shoots and twigs), the relatively low MC-values of our spring samples of *Q. suber*, combined with high flammability [6,15,88], made this species moderately flammable, in accordance with Henaoui [93].

(ii) Low volatile compound content of leaves: *P. lentiscus* [91], which resulted in slow burning; *A. unedo* [91], particularly regarding twig flammability (mainly involving leaves), as shown in our findings where this species was found to be the least flammable since flammability increases with volatile compound content [94–96].
(iii) high ash content: *P. lentiscus* [90]; *Cistus salviifolius* [91].
(iv) Low SVR: *P. lentiscus*, for which the flammability classification can be explained by the SVR of its leaves, was found to be the lowest among all species examined [61]; *C. salviifolius*, whose flammability varied according to the flammability-testing experiment used (shoot- or twig-flammability), as stems had higher SVR but leaves had lower SVR.
(v) long TI: offers an additional explanation for the low flammability of *P. lentiscus* [6,90]. TI is the best descriptor of ignitability [35] and is negatively correlated with combustibility and sustainability [35,38,54].

For twig flammability, samples were 1g-terminal twigs with their leaves, which are fine fuel, while for shoot flammability, samples were 70 cm-long shoots containing large amount of coarse fuel. Fine fuel have higher SVR than coarse fuel, making their flame residence time shorter, decreasing BT [87]. However, high SVR of fine fuel increases TI [9,15,19,84].The twig-flammability experimental test involved MC, which was significantly and negatively correlated with FLT. Additionally, MC was almost orthogonal to shoot flammability traits (BT, MT, and BL), with large angles between them, indicating a negative correlation between MC and FLS. This demonstrates similar behaviour across both flammability-testing experiments regarding MC. These findings are supported by the strong correlation observed between the rankings derived from FLS and an expert opinion-based flammability list used by Wyse et al. [7]. However, this list was found to be uncorrelated with the leaf-level flammability ranking [15], a difference attributed to the tested fuel levels (twig- or shoot-level flammability) [15] and the scale considered [57,58]. The traits measured in our experiments are also different, have different units and are combined differently to create their respective indices with distinct scales ([0; 20] for FLT and [0; 3] for FLS), although the results were quite similar.

It turns out that both flammability testing experiments, although they examine the flammability issue at different scales (field *versus* laboratory) and plant levels (shoot *versus* twig), have classified the same species into two main groups: “Most flammable” (*P. canariensis*) and “Less flammable” (all other species). However, the shoot-flammability experimental test provided more detail by distinguishing between the four less flammable species than *P. canariensis*. This consistent classification in the study site for the five species studied aligns with several studies showing that field results often match laboratory findings, provided that the leaf physical or biochemical traits of the plant samples are not altered [12]. For example, in a study comparing the flammability of undisturbed litter samples from the field with disturbed samples handled in the laboratory, the results were similar in terms of time-to-ignition and flaming duration [97]. In another study, comparable flammability was observed at both laboratory and field scales for samples of smoothed and textured corkback under moderate fire severity conditions [61].

However, further insights could be gained by incorporating the heat release rate (HRR) and effective heat of combustion (EHC), which are now recognised as essential for capturing the energy dynamics of combustion [26,27,35,98]. Future studies should include oxygen consumption calorimetry to compare the combustion efficiency of different species and to improve the predictive power of flammability indices. Specifically, integrating HRR and EHC measurements via oxygen consumption calorimetry (as in Babrauskas [26,27,98]) would allow direct comparisons of combustion efficiency across various species and help resolve discrepancies observed between laboratory- scale and field- scale flammability indices. Such integration would also provide a stronger thermodynamic basis for interpreting empirical flammability rankings, especially when translating laboratory outcomes into predictive indicators of wildfire behaviour in natural ecosystems. Overall, the metrics employed in both flammability testing experiments can quantify key flammability attributes, namely ignitability, combustibility, sustainability, and consumability. For shoot flammability, combustibility was assessed using MT [35,38] and BT [35], with BT being strongly correlated with HRR and thus capable of replacing it [35]. As an indicator of combustibility [36], EHC can itself be evaluated through MT and BT [35,38]. Sustainability was measured *via* BT [9,34,35,99,100] and consumability through BL [35]. For twig flammability, ignitability was expressed through TI [35,101,102]. Besides their widespread use in ranking plant flammability [4,33,102,103], TI is a proxy for HRR, as plants that are more ignitable (*i.e*., lower TI) tend to have higher HRR [35]. This correlation was also confirmed during field burnings, where more ignitable vegetation produced larger flames [54]. Furthermore, combustibility metrics can offer insights into EHC [36], while FH, MT, and BT are good indicators of combustibility [4,9,35,38,104]; the latter metrics can also serve as proxies for EHC. FH is also considered indicative of sustainability [35]. TI and FH are suitable for both field- [54–56] and laboratory- scale fire experiments [23]. As demonstrated, the metrics used in the two flammability testing experiments discussed in our study complement each other and are applicable in both field and laboratory settings. This enhances the robustness of our classification approach, particularly when technical resources are limited.

The consistency of the shoot flammability method, in the study site for the five species studied, was reinforced by the findings of Dent et al. [39], where the measured shoot flammability components were positively correlated with the proportion of dead material. The low MC of dead material means less energy is required to initiate combustion [39]. Shoot flammability is particularly interesting because it is largely correlated with phylogenetic relatedness [105,106] and high flammability may result from parallel evolution driven by environmental factors [105–107], such as fire regimes [105]. It can primarily be predicted using phylogeny, growth form (forbs, grasses, shrubs and trees) [105] and the susceptibility of the habitat to recurrent fires (fire-proneness) [105,106]. Conversely, the twig-flammability experimental test, which is a laboratory-scale test, is simpler and more practical, as the required device is straightforward [33] and fire tests are quicker, although the number of replicates (36) is higher for Hachmi et al. [34] and Essaghi et al. [6]. Twig-level flammability tests remain widely used [19,22,30,32,23,20,108], mostly with the same device employed in the present work [23].

Overall, given that laboratory tests can only be conducted on a limited scale, such as cut material, and that sample exposure to heat is not comparable to wildfire conditions (which involve undisturbed plant material) [37], the flammability of cut plant parts has been considered a poor predictor of the flammability of whole plants (undisturbed material), litter beds [11] and canopy [11,109]. However, heavy reliance on laboratory–scale flammability tests has been driven by the challenges in measuring flammability in wildland fires [12,44]. In reality, field-scale fire experiments are often constrained by landscape and economic impacts [59], as well as authorisation and safety regulations [22,37,44]. Additionally, the difficulties of standardised measurements in field conditions hinder the use of field-scale flammability tests [44]. Nevertheless, because fire impacts occur at different scales, fire behaviour can be assessed using various flammability metrics, which require testing both cut and undisturbed samples [43,44] and probably using dynamic heat flux to replicate an approaching fire front, since dynamic and static heat flux showed significantly different results [25]. Flammability metrics for cut material should correspond to those of undisturbed material in the field to facilitate scaling and accurately reflect ground realities [37]. Therefore, by measuring flammability on small cut samples, each trait’s contribution to fire risk can be evaluated, making these tests valuable for calibrating fire behaviour prediction models [44]. Furthermore, laboratory experiments on small samples (cut samples) are justified and objective because natural selection and genetic control influence individual traits, which necessitate standardised flammability measurements on leaves or small branches under controlled conditions [44].

However, the positions of the fuel and fire are among the main challenges for the realism of flammability-testing experiments based on cut plant samples [59]. In a study aiming to determine the stem and cork characteristics related to the likelihood of reaching the lethal cambial temperature, significant differences between cut samples (lab) and undisturbed ones (field conditions) were observed in (i) the time to reach the lethal cambial temperature of cork [59], (ii) the emission of highly volatile isoprenoids and heat [37,110], and (iii) the percentage of samples reaching lethal cambial temperature under severe fire conditions [59]. Our approach, being simple, easy, and practical, using accessible material, can produce interesting outcomes that align with the existing literature. However, the flammability classification of species needs to be complemented by additional flammability assessment methods comparing the two studied experimental tests and involving more plant species. Field burnings under standardised conditions should also be tested and compared to the two flammability experiments conducted in this work. This is a partial result that have to be tested in other sites using different provenances and additional species for a future generalisation.

## Conclusion

Fire risk assessment, using various approaches to flammability rating, is essential for effective wildfire prevention. These methods present challenges in realism and objectivity, aiding in selecting appropriate afforestation species and improving fire behaviour prediction systems. A common debate at the plant level during flammability tests involves comparing twig *versus* shoot, reflecting the scale of fire experiments (laboratory *versus* field). Although the fire experiments tested yielded consistent outcomes which would pave the way for a realistic flammability assessment method based on available technical resources, some methodological improvements need to be emphasised: (i) TI and MC may represent informative complementary traits to interpret shoot-level flammability due to their significant impact on flammability; (ii) dynamic (increasing) heat flux replicating an approaching fire front have to be tested for both studied fire experiments; (iii) quantification of the radiant heat flux for the grill used to measure shoot flammability in order to enable energy-normalised comparisons. The comparison of the studied experiments needs to be tested in more sites in Morocco and elsewhere and using wider range of species to obtain the generalisability of the results. Additional techniques involving broader parameters and larger-scale field studies should be testedin order to offer a clearer understanding of flammability trends. Such research should also focus on applying more physically meaningful descriptors, like HRR and EHC, to improve cross-species comparisons, thereby increasing the predictive accuracy of plant flammability indices and ultimately enhancing fire behaviour prediction systems.

## Supporting information

S1 Table Tukey’s pairwise comparison test between the studied species for each flammability metric and the resulting flammability index for twig and shoot flammability. This supporting information includes (i) Table 1 where are presented the results of the Tukey’s pairwise comparison test used to see how statistically different are the studied species from each other’s in terms of shoot flammability metrics, and (ii) Table 2 where are presented the results of the Tukey’s pairwise comparison test used to see how statistically different are the studied species from each other’s in terms of twig flammability metrics.
(PDF)
