# Supplementary material for: Assessing the consistency of flammability indices across field and laboratory experimental tests for some Moroccan forest fuels
Source: PLoS One. 2026 Apr 2;21(4):e0345668. doi: 10.1371/journal.pone.0345668 (PMC13046143; doi:10.1371/journal.pone.0345668)
Supplement: S1 Table — This supporting information includes (i) Table 1 where are presented the results of the Tukey’s pairwise comparison test used to see how statistically different are the studied species from each other’s in terms of shoot flammability metrics, and (ii) Table 2 where are presented the results of the Tukey’s pairwise comparison test used to see how statistically different are the studied species from each other’s in terms of twig flammability metrics. (PDF) [file pone.0345668.s001.pdf]

S1 Table 1 : Supporting information for Table 1.

Multiple comparisons							
Tukey’s test							
						Confidence interval at 95%	
Dependent variable	(I) Species	(J) Species	Means difference (I-J)	Standard error	Signification	Lower bound	Upper bound
BT	A unedo	C salviifolius	104,7000	266,0983	0,995	-688,017	897,417
		P canariensis	-831,6000	283,6617	0,055	-1676,639	13,439
		P lentiscus	,9000	253,7147	1,000	-754,926	756,726
		Q suber	-96,4200	266,0983	0,996	-889,137	696,297
	C salviifolius	A unedo	-104,7000	266,0983	0,995	-897,417	688,017
		P canariensis	-936,3000*	294,7899	0,033	-1814,490	-58,110
		P lentiscus	-103,8000	266,0983	0,995	-896,517	688,917
		Q suber	-201,1200	277,9306	0,949	-1029,086	626,846
	P canariensis	A unedo	831,6000	283,6617	0,055	-13,439	1676,639
		C salviifolius	936,3000*	294,7899	0,033	58,110	1814,490
		P lentiscus	832,5000	283,6617	0,055	-12,539	1677,539
		Q suber	735,1800	294,7899	0,130	-143,010	1613,370
	P lentiscus	A unedo	-,9000	253,7147	1,000	-756,726	754,926
		C salviifolius	103,8000	266,0983	0,995	-688,917	896,517
		P canariensis	-832,5000	283,6617	0,055	-1677,539	12,539
		Q suber	-97,3200	266,0983	0,996	-890,037	695,397
	Q suber	A unedo	96,4200	266,0983	0,996	-696,297	889,137
		C salviifolius	201,1200	277,9306	0,949	-626,846	1029,086
		P canariensis	-735,1800	294,7899	0,130	-1613,370	143,010
		P lentiscus	97,3200	266,0983	0,996	-695,397	890,037
MT	A unedo	C salviifolius	1,2367	7,2516	1,000	-20,366	22,840
		P canariensis	-42,4083*	7,7303	0,000	-65,437	-19,380
		P lentiscus	6,0667	6,9142	0,902	-14,531	26,664
		Q suber	-5,4033	7,2516	0,943	-27,006	16,200

	C salviifolius	A unedo	-1,2367	7,2516	1,000	-22,840	20,366
		P canariensis	-43,6450*	8,0335	0,000	-67,577	-19,713
		P lentiscus	4,8300	7,2516	0,962	-16,773	26,433
		Q suber	-6,6400	7,5741	0,902	-29,203	15,923
	P canariensis	A unedo	42,4083*	7,7303	0,000	19,380	65,437
		C salviifolius	43,6450*	8,0335	0,000	19,713	67,577
		P lentiscus	48,4750*	7,7303	0,000	25,446	71,504
		Q suber	37,0050*	8,0335	0,001	13,073	60,937
	P lentiscus	A unedo	-6,0667	6,9142	0,902	-26,664	14,531
		C salviifolius	-4,8300	7,2516	0,962	-26,433	16,773
		P canariensis	-48,4750*	7,7303	0,000	-71,504	-25,446
		Q suber	-11,4700	7,2516	0,524	-33,073	10,133
	Q suber	A unedo	5,4033	7,2516	0,943	-16,200	27,006
		C salviifolius	6,6400	7,5741	0,902	-15,923	29,203
		P canariensis	-37,0050*	8,0335	0,001	-60,937	-13,073
		P lentiscus	11,4700	7,2516	0,524	-10,133	33,073
BL	A unedo	C salviifolius	9,0333	6,2493	0,607	-9,583	27,650
		P canariensis	-23,9167*	6,6617	0,013	-43,762	-4,071
		P lentiscus	16,3333	5,9584	0,081	-1,417	34,084
		Q suber	-3,9667	6,2493	0,968	-22,583	14,650
	C salviifolius	A unedo	-9,0333	6,2493	0,607	-27,650	9,583
		P canariensis	-32,9500*	6,9231	0,001	-53,574	-12,326
		P lentiscus	7,3000	6,2493	0,769	-11,317	25,917
		Q suber	-13,0000	6,5271	0,304	-32,445	6,445
	P canariensis	A unedo	23,9167*	6,6617	0,013	4,071	43,762
		C salviifolius	32,9500*	6,9231	0,001	12,326	53,574
		P lentiscus	40,2500*	6,6617	0,000	20,404	60,096
		Q suber	19,9500	6,9231	0,061	-,674	40,574
	P lentiscus	A unedo	-16,3333	5,9584	0,081	-34,084	1,417
		C salviifolius	-7,3000	6,2493	0,769	-25,917	11,317

		P canariensis	-40,2500*	6,6617	0,000	-60,096	-20,404
		Q suber	-20,3000*	6,2493	0,028	-38,917	-1,683
	Q suber	A unedo	3,9667	6,2493	0,968	-14,650	22,583
		C salviifolius	13,0000	6,5271	0,304	-6,445	32,445
		P canariensis	-19,9500	6,9231	0,061	-40,574	,674
		P lentiscus	20,3000*	6,2493	0,028	1,683	38,917
BB	A unedo	C salviifolius	12,9048	8,9275	0,607	-13,691	39,500
		P canariensis	-34,1667*	9,5168	0,013	-62,517	-5,816
		P lentiscus	23,3333	8,5120	0,081	-2,024	48,691
		Q suber	-5,6667	8,9275	0,968	-32,262	20,929
	C salviifolius	A unedo	-12,9048	8,9275	0,607	-39,500	13,691
		P canariensis	-47,0714*	9,8901	0,001	-76,534	-17,608
		P lentiscus	10,4286	8,9275	0,769	-16,167	37,024
		Q suber	-18,5714	9,3245	0,304	-46,349	9,207
	P canariensis	A unedo	34,1667*	9,5168	0,013	5,816	62,517
		C salviifolius	47,0714*	9,8901	0,001	17,608	76,534
		P lentiscus	57,5000*	9,5168	0,000	29,149	85,851
		Q suber	28,5000	9,8901	0,061	-,963	57,963
	P lentiscus	A unedo	-23,3333	8,5120	0,081	-48,691	2,024
		C salviifolius	-10,4286	8,9275	0,769	-37,024	16,167
		P canariensis	-57,5000*	9,5168	0,000	-85,851	-29,149
		Q suber	-29,0000*	8,9275	0,028	-55,595	-2,405
	Q suber	A unedo	5,6667	8,9275	0,968	-20,929	32,262
		C salviifolius	18,5714	9,3245	0,304	-9,207	46,349
		P canariensis	-28,5000	9,8901	0,061	-57,963	,963
		P lentiscus	29,0000*	8,9275	0,028	2,405	55,595
BR	A unedo	C salviifolius	-,06139	,03813	0,507	-,1750	,0522
		P canariensis	,06373	,04064	0,533	-,0573	,1848
		P lentiscus	,06794	,03635	0,363	-,0404	,1762

		Q suber	-0,00472	,03813	1,000	-,1183	,1089
	C salviifolius	A unedo	,06139	,03813	0,507	-,0522	,1750
		P canariensis	0,12511	,04224	0,052	-,0007	,2509
		P lentiscus	,12932*	,03813	0,021	,0157	,2429
		Q suber	0,05666	,03982	0,620	-,0620	,1753
	P canariensis	A unedo	-0,06373	,04064	0,533	-,1848	,0573
		C salviifolius	-0,12511	,04224	0,052	-,2509	,0007
		P lentiscus	0,00421	,04064	1,000	-,1169	,1253
		Q suber	-0,06845	,04224	0,501	-,1943	,0574
	P lentiscus	A unedo	-0,06794	,03635	0,363	-,1762	,0404
		C salviifolius	-0,12932*	,03813	0,021	-,2429	-,0157
		P canariensis	-,00421	,04064	1,000	-,1253	,1169
		Q suber	-,07266	,03813	0,345	-,1862	,0409
	Q suber	A unedo	,00472	,03813	1,000	-,1089	,1183
		C salviifolius	-,05666	,03982	0,620	-,1753	,0620
		P canariensis	,06845	,04224	0,501	-,0574	,1943
		P lentiscus	,07266	,03813	0,345	-,0409	,1862
FLS	A unedo	C salviifolius	,05220	,06231	0,916	-,1334	,2378
		P canariensis	-,24331*	,06642	0,011	-,4412	-,0454
		P lentiscus	,24674*	,05941	0,004	,0698	,4237
		Q suber	-,03775	,06231	0,973	-,2234	,1479
	C salviifolius	A unedo	-,05220	,06231	0,916	-,2378	,1334
		P canariensis	-,29551*	,06903	0,003	-,5011	-,0899
		P lentiscus	,19454*	,06231	0,037	,0089	,3802
		Q suber	-,08995	,06508	0,645	-,2838	,1039
	P canariensis	A unedo	,24331*	,06642	0,011	,0454	,4412
		C salviifolius	,29551*	,06903	0,003	,0899	,5011
		P lentiscus	,49005*	,06642	0,000	,2922	,6879
		Q suber	,20556	,06903	0,050	-,0001	,4112
	P lentiscus	A unedo	-,24674*	,05941	0,004	-,4237	-,0698
		C salviifolius	-,19454*	,06231	0,037	-,3802	-,0089

		P canariensis	-,49005*	,06642	0,000	-,6879	-,2922
		Q suber	-,28449*	,06231	0,001	-,4701	-,0989
	Q suber	A unedo	,03775	,06231	0,973	-,1479	,2234
		C salviifolius	,08995	,06508	0,645	-,1039	,2838
		P canariensis	-,20556	,06903	0,050	-,4112	,0001
		P lentiscus	,28449*	,06231	0,001	,0989	,4701

*Means difference is significant at 0.05.

S1 Table 2: Supporting information for Table 2.

Multiple comparisons							
Tukey's test							
Dependent variable	(I) Species	(J) Species	Means difference (I-J)	Standard error	Signification	Confidence interval at 95%	
						Lower bound	Upper bound
TI	A unedo	C salviifolius	1,56748	,61542	0,085	-,1291	3,2641
		P canariensis	5,22889*	,61107	0,000	3,5443	6,9135
		P lentiscus	,92361	,61107	0,556	-,7610	2,6082
		Q suber	,97034	,61542	0,514	-,7262	2,6669
	C salviifolius	A unedo	-1,56748	,61542	0,085	-3,2641	,1291
		P canariensis	3,66140*	,61542	0,000	1,9648	5,3580
		P lentiscus	-,64387	,61542	0,833	-2,3404	1,0527
		Q suber	-,59714	,61974	0,871	-2,3056	1,1113
	P canariensis	A unedo	-5,22889*	,61107	0,000	-6,9135	-3,5443
		C salviifolius	-3,66140*	,61542	0,000	-5,3580	-1,9648
		P lentiscus	-4,30528*	,61107	0,000	-5,9899	-2,6207
		Q suber	-4,25855*	,61542	0,000	-5,9551	-2,5620
	P lentiscus	A unedo	-,92361	,61107	0,556	-2,6082	,7610
		C salviifolius	,64387	,61542	0,833	-1,0527	2,3404
		P canariensis	4,30528*	,61107	0,000	2,6207	5,9899
		Q suber	,04673	,61542	1,000	-1,6498	1,7433
	Q suber	A unedo	-,97034	,61542	0,514	-2,6669	,7262
		C salviifolius	,59714	,61974	0,871	-1,1113	2,3056
		P canariensis	4,25855*	,61542	0,000	2,5620	5,9551
		P lentiscus	-,04673	,61542	1,000	-1,7433	1,6498
FH	A unedo	C salviifolius	,0520	1,0281	1,000	-2,782	2,886
		P canariensis	-15,0139*	1,0208	0,000	-17,828	-12,200
		P lentiscus	-10,9444*	1,0208	0,000	-13,759	-8,130
		Q suber	-12,3337*	1,0281	0,000	-15,168	-9,500

	C salviifolius	A unedo	-,0520	1,0281	1,000	-2,886	2,782
		P canariensis	-15,0659*	1,0281	0,000	-17,900	-12,232
		P lentiscus	-10,9964*	1,0281	0,000	-13,831	-8,162
		Q suber	-12,3857*	1,0353	0,000	-15,240	-9,532
	P canariensis	A unedo	15,0139*	1,0208	0,000	12,200	17,828
		C salviifolius	15,0659*	1,0281	0,000	12,232	17,900
		P lentiscus	4,0694*	1,0208	0,001	1,255	6,884
		Q suber	2,6802	1,0281	0,073	-,154	5,514
	P lentiscus	A unedo	10,9444*	1,0208	0,000	8,130	13,759
		C salviifolius	10,9964*	1,0281	0,000	8,162	13,831
		P canariensis	-4,0694*	1,0208	0,001	-6,884	-1,255
		Q suber	-1,3893	1,0281	0,659	-4,223	1,445
	Q suber	A unedo	12,3337*	1,0281	0,000	9,500	15,168
		C salviifolius	12,3857*	1,0353	0,000	9,532	15,240
		P canariensis	-2,6802	1,0281	0,073	-5,514	,154
		P lentiscus	1,3893	1,0281	0,659	-1,445	4,223
FLT	A unedo	C salviifolius	-,61379	,31047	0,282	-1,4697	,2421
		P canariensis	-3,61194*	,30828	0,000	-4,4618	-2,7621
		P lentiscus	-,74184	,30828	0,118	-1,5917	,1080
		Q suber	-,82081	,31047	0,067	-1,6767	,0351
	C salviifolius	A unedo	,61379	,31047	0,282	-,2421	1,4697
		P canariensis	-2,99816*	,31047	0,000	-3,8540	-2,1423
		P lentiscus	-,12805	,31047	0,994	-,9839	,7278
		Q suber	-,20702	,31265	0,964	-1,0689	,6549
	P canariensis	A unedo	3,61194*	,30828	0,000	2,7621	4,4618
		C salviifolius	2,99816*	,31047	0,000	2,1423	3,8540
		P lentiscus	2,87011*	,30828	0,000	2,0203	3,7199
		Q suber	2,79113*	,31047	0,000	1,9352	3,6470
	P lentiscus	A unedo	,74184	,30828	0,118	-,1080	1,5917
		C salviifolius	,12805	,31047	0,994	-,7278	,9839
		P canariensis	-2,87011*	,30828	0,000	-3,7199	-2,0203
		Q suber	-,07897	,31047	0,999	-,9349	,7769
	Q suber	A unedo	,82081	,31047	0,067	-,0351	1,6767
		C salviifolius	,20702	,31265	0,964	-,6549	1,0689
		P canariensis	-2,79113*	,31047	0,000	-3,6470	-1,9352
		P lentiscus	,07897	,31047	0,999	-,7769	,9349

*Means difference is significant at 0.05.
